# Gamete compatibility genes in mammals: candidates, applications and a potential path forward

**DOI:** 10.1098/rsos.170577

**Published:** 2017-08-30

**Authors:** Leah Springate, Timothy R. Frasier

**Affiliations:** Department of Biology, Saint Mary's University, 923 Robie St, Halifax, Nova Scotia, Canada B3H 3C3

**Keywords:** cryptic female choice, gamete compatibility, mate compatibility, post-copulatory sexual selection

## Abstract

Fertilization represents a critical stage in biology, where successful alleles of a previous generation are shuffled into new arrangements and subjected to the forces of selection in the next generation. Although much research has been conducted on how variation in morphological and behavioural traits lead to variation in fertilization patterns, surprisingly little is known about fertilization at a molecular level, and specifically about how genes expressed on the sperm and egg themselves influence fertilization patterns. In mammals, several genes have been identified whose products are expressed on either the sperm or the egg, and which influence the fertilization process, but the specific mechanisms are not yet known. Additionally, in 2014 an interacting pair of proteins was identified: ‘Izumo’ on the sperm, and ‘Juno’ on the egg. With the identification of these genes comes the first opportunity to understand the molecular aspects of fertilization in mammals, and to identify how the genetic characteristics of these genes influence fertilization patterns. Here, we review recent progress in our understanding of fertilization and gamete compatibility in mammals, which should provide a helpful guide to researchers interested in untangling the molecular mechanisms of fertilization and the resulting impacts on population biology and evolutionary processes.

## 1. Introduction

The process of fertilization represents one of the most important steps in population biology and evolution: being the stage at which successful alleles of a previous generation are shuffled into new combinations and packaged as individuals to be subjected to the forces of selection in the next generation. Factors that influence patterns of fertilization have subsequent impacts on the variance in reproductive performance across individuals, which ultimately influences the reproductive and growth potential of a population [1]. Moreover, factors that divide a population into groups of individuals where intra-group fertilization occurs more readily than inter-group fertilization can ultimately lead to speciation [2]. Given these wide-ranging effects, improving our understanding of the forces shaping patterns of fertilization is a goal of biologists across a broad range of specialties. Although much research has been conducted on how variations in morphological and behavioural traits lead to variation in fertilization patterns [3–5], surprisingly little is known about how genes expressed on the sperm and the eggs themselves—and which represent the crucial ‘locks and keys’ needed for successful fertilization—influence fertilization success and fitness.

Fertilization is mediated by a complex series of interactions between the sperm and the egg, key steps of which require complementary interactions between proteins expressed on the surface of each gamete [6–8]. The properties of these proteins influence how compatible a sperm and egg are, in terms of potential for successful fertilization, which is often referred to as their ‘gametic compatibility’. Until recently, the complementary genes controlling this compatibility had only been well described and studied in a few marine invertebrates, most notably abalone [9–12] and urchins [13–16]. In both taxa, the genotype of the receptor on the egg directly determines which sperm genotypes are capable of fertilization [9,12,14]. These sperm–egg interactions, and resulting non-random fertilization patterns, scale up to having large impacts on patterns of individual reproductive success within populations [14], and on the development of reproductive barriers during speciation [16–19]. The complementary nature of these genes means that there is not one ‘best’ genotype, but rather what genotype is ‘good’ depends on the genotype of the corresponding gamete. This sort of epistasis, or non-additive interaction between alleles, provides an important mechanism for maintaining genetic diversity within populations and for providing the raw material to drive reproductive isolation and speciation through the presence of segregating incompatible alleles [20]. Although the benefits of genes influencing gamete compatibility are clear in species with external fertilization such as these, to ensure eggs are fertilized by sperm of the correct species, much evidence exists that such genes are important also in organisms with internal fertilization, such as mammals.

Genes involved in reproduction, and in gamete compatibility in particular, have proved to be among the fastest evolving genes in organisms yet studied (along with those of the immune system) [11,21,22]. There are three primary hypotheses, which are not mutually exclusive, regarding the underlying selection pressures [21]. The first is sperm competition, where the genetic complement of a sperm could influence its chances of fertilization success at many stages throughout the fertilization process, and intense competition among sperm could then lead to rapid evolution of the associated genes. Second is sexual selection, where particular sperm–egg combinations have higher success rates than others, leading to the continual coevolution of genes expressed on the gametes of both sexes. Last is sexual conflict, where selection on eggs to block polyspermy, and intense competition among sperm, provide conflicting selection pressures on the gametes (on eggs to make multiple fertilization difficult, and on sperm to more rapidly fertilize the egg). This process would lead to a continual coevolution of the genes involved in such strategies. Related to the ‘sexual selection’ hypothesis is the rapid divergence in gamete compatibility genes often found between recently diverged species [17,19,23]. Selection against cross-species fertilization could lead to rapid divergence of gamete compatibility genes, relative to other parts of the genome, and thus lead to effective reproductive boundaries between taxa.

In mammals, many genes have been identified whose products are expressed on either the sperm or the egg, and are somewhat involved in gamete compatibility, but the specific interactions and mechanisms are not yet known [22,24–28]. However, this changed in 2014 when a pair of genes (called ‘Izumo’ for the sperm surface protein, and ‘Juno’ for the complementary egg receptor) was identified with a specific ligand–receptor relationship [29,30]. With the identification of these genes comes the first opportunity to understand the details and mechanisms of gamete compatibilities in mammals, to identify how genetic variation at these genes influence fertilization patterns and fitness, and to assess the subsequent implications for the development of reproductive barriers and speciation.

Given this recent progress, it seems timely to review our state of knowledge of these candidate genes and the processes that they influence. Here, we provide a brief overview of the structure of mammalian gametes and the fertilization process in mammals, review what is known about key candidate genes involved and provide a brief review of the key areas where the analysis of such genes may be fruitful. The hope is that such a review will provide motivation, as well as a guide, for researchers interested in untangling the mechanisms of gamete compatibility and the resulting impacts on population biology and evolutionary processes.

## 2. Gamete structure

### 2.1. The egg

**Figure 1.**
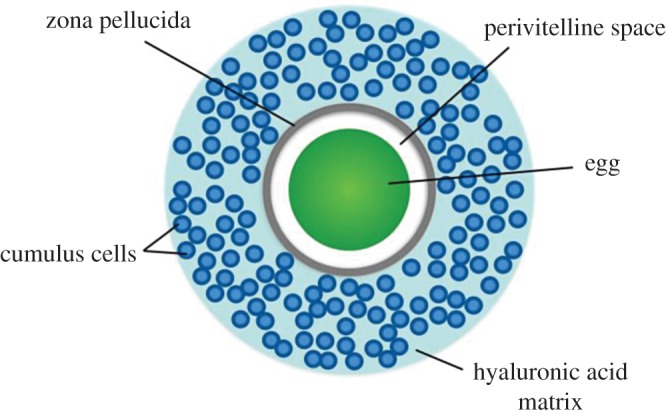
Overview of egg structure. The ovulated egg is surrounded by a hyaluronic acid matrix, which contains cumulus cells. The zona pellucida (ZP) separates the cumulus cells from the egg. The perivitelline space is the space between the ZP and the membrane of the egg. Figure drawn from [8,31].

An ovulated mammalian egg is surrounded by two key layers (figure 1). The first is an outer layer of cumulus cells that are contained in an extracellular matrix composed mainly of hyaluronic acid (also called the cumulus oophorus) [8,32]. Cumulus cells promote oocyte growth and development, and secrete progesterone, which is probably one of the chemoattractants—attracting the sperm to the egg [31,33]. The cumulus oophorus has many soluble factors and hormones that affect the egg and sperm in several ways, such as coordinating oocyte maturation and transport, and stimulating sperm motility [32]. The cumulus cells also act as the first of several barriers to spermatozoa, representing a dense mass through which only sperm that have undergone proper initial steps (see below) can pass. Interestingly, the cumulus layer is shed shortly after ovulation in marsupials and monotremes, and is therefore only a key player in fertilization within eutherian mammals [34,35]

The second major layer is a thick glycoprotein layer called the zona pellucida (ZP) [7]. It often serves as a species-selective barrier for sperm, and the binding of sperm to the ZP represents the first (of two) major interactions between the sperm and the egg. The ZP is composed of three different glycoproteins in most mammals: ZP1, ZP2 and ZP3; but humans and other primates have an additional glycoprotein, ZP4 [36,37]. Alterations to the ZP after fertilization prevent polyspermy and protect the early-stage embryo [35].

Interior to the ZP is the perivitelline space, which separates the egg proper from the zona pellucida, creating an area of protection [38]. Lastly, the egg is surrounded by a plasma membrane, to which spermatozoa bind during fertilization.

### 2.2. The sperm

Mammalian sperm can be divided into three main sections: the head, midpiece and tail, with lengths and characteristics that vary across species [39]. The sperm head contains the nucleus and the acrosome. The nucleus contains the haploid genome needed for fertilization. The acrosome is a secretory organelle that covers the first two-thirds of the sperm head, and is key to the binding of spermatozoa to the egg (figure 3) [39,40]. The midpiece is the central segment that connects the tail to the sperm head. It contains a central filamentous core surrounded by a large number of mitochondria as energy suppliers for the spermatozoa. The tail, or flagellum, is the longest part of the sperm, and is responsible for propulsion to the site of the egg [39].

## 3. Fertilization

Fertilization is an extremely complex, multistep process of which many details remain poorly understood. For our purposes, we will consider the processes involved in fertilization that occur post copulation. This delineation point is arbitrary, and prior aspects of reproduction such as the structure of reproductive organs, and mating systems and behaviour, obviously influence patterns of fertilization. However, in contrast to the wide variation in these characteristics across mammals, there is much similarity in the process once sperm have entered the female reproductive tract. Therefore, this stage serves as a suitable starting point for examining the context and processes associated with the molecular aspects of fertilization.

When sperm first enter the reproductive tract, two main objectives can be envisaged: evading the female immune system and targeting movement towards the egg. The importance of the former can be seen from data where seminal fluid triggers an invasion of antisperm antibodies and white blood cells into the vagina [41–43] that can proceed to break down the spermatozoa (motile sperm) [44,45]. Indeed, it is thought that avoiding such an immune response is why many species evolved genitalia capable of depositing spermatozoa directly into the uterus, or at least close to the cervix where they can then quickly be moved through the cervix into the uterus [46]. Components within the seminal plasma also appear to provide at least some additional protection from phagocytosis [47]. This immune response, and the medium of the cervical mucus, are thought to limit the progress of a large portion of the sperm (including those that are malformed or damaged), whereas a small portion of morphologically normal sperm may proceed rapidly into the oviduct.

**Figure 2.**
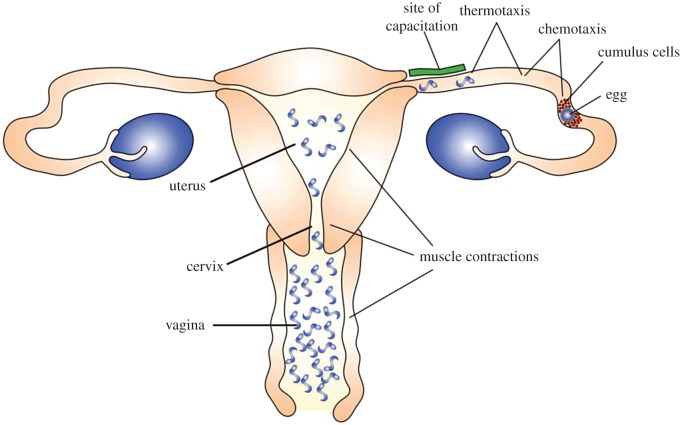
Overview of major structures, sites and transport/guiding processes involved in fertilization. Although there is variation across mammals, this is meant to represent generic features of mammalian reproduction. Figure drawn from [49,51].

Numerous factors appear to aid the movement of sperm towards the egg, and the relative importance of each may vary across species. In general, four main factors are key [46,48,49]. First, uterine contractions can efficiently move large numbers of sperm through the initial components of the reproductive tract (vagina, cervix, uterus). Second, folds present in the tissues may serve as pathways directing sperm through the cervix and uterus, and towards the oviduct. Third, thermotaxis—or the movement of spermatozoa along a temperature gradient—helps guide sperm down the fallopian tube towards the site of fertilization, which is 1–2° warmer than the entrance of the fallopian tube [49,50]. The fourth, and perhaps most interesting, factor is chemoattraction: where sperm are attracted by chemical signals released from the egg [48,51]. Such a process is prevalent in the animal kingdom, but less well understood in mammals. For example, the specific chemicals used have been identified for many non-mammalian species, but have yet to be identified in mammals [49,51] (figure 2).

**Figure 3.**
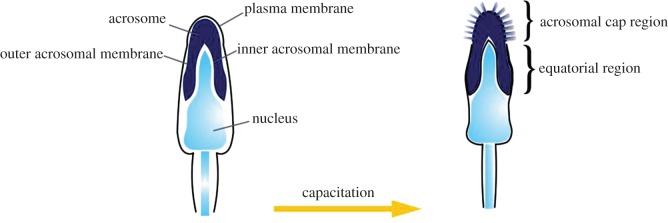
Sperm structure and capacitation. Diagram of the sperm head containing the nucleus with the haploid genome, and the acrosome, which is a secretory organelle. The acrosome has two membranes, an inner and outer. Capacitation causes multiple physiological changes in the head, acrosome and tail of the sperm, which is necessary for fertilization. Figure drawn from reference [8].

### 3.1. Modification of sperm prior to fertilization

Successful movement of the gametes towards one another is not the only hurdle to be overcome for fertilization to be successful. Instead, early studies showed that spermatozoa cannot fertilize eggs immediately after ejaculation, but rather require an incubation time in the female reproductive tract before acquiring this potential [52–55]. These studies provided early indications of the complexity of fertilization, and the important role of environmental conditions within the female reproductive tract [52,55]. Although such an incubation time is required across mammals, the necessary length of time varies across species, ranging from approximately 1 h in humans to approximately 5 h in rabbits and cows [56–58]. These data demonstrate that the sperm must undergo modifications that are triggered by the environment in order to interact properly with the egg. Two such transitions are now known to occur: capacitation and the acrosome reaction.

#### 3.1.1. Capacitation

As spermatozoa make their way through the uterus and into the oviductal isthmus, they become reversibly bound to the oviductal epithelium [51]. This is the stage at which spermatozoa become capacitated (figure 2). One of the key steps that appears to trigger this process is the removal (*in vitro*) or dilution (*in vivo*) of the seminal fluid associated with the spermatozoa, which is a known inhibitor of capacitation [55,59,60]. Not all spermatozoa undergo capacitation at the same time, however, and at any given time only a small portion (approx. 10%) are capacitated, with a relatively high turnover rate of which sperm are capacitated and which are not [55].

The processes that take place during capacitation have two major effects on the fertilization abilities of the sperm. First, it is at this time when sperm become ‘hyperactivated’. Hyperactivation usually involves increased amplitude and asymmetry in flagellar beating patterns, and appears necessary for the spermatozoa to break free from their bonds with the oviductal epithelium, complete their journey towards the egg and penetrate the outer layers of the egg [61,62]. Second, it is during capacitation when the proteins needed for sperm–egg interactions become ‘unmasked’ due to the removal and/or changes in the proteins present on the plasma membrane on the head of the sperm [55,63,64] (figure 3). Thus, it is at this stage when the first proteins involved in sperm–egg interaction are exposed, and the genes underlying such proteins should be a key target in investigations into the molecular aspects of gametic compatibility.

After capacitation, spermatozoa move through the fallopian tube towards the egg, probably guided by a combination of thermotaxis, chemotaxis and oviductal contractions. Only capacitated sperm can make their way through the cumulus cells surrounding the egg [55].

#### 3.1.2. The acrosome reaction

**Figure 4.**
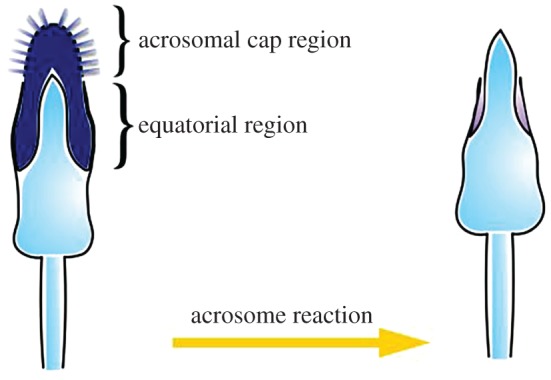
Changes to spermatozoa during the acrosome reaction. The inner acrosomal membrane is exposed allowing the spermatozoa to bind to and penetrate the zona pellucida, and to bind to the egg plasma membrane. Figure drawn from [8,37].

The second major transition that must take place in the spermatozoa for fertilization to be successful is the acrosome reaction (AR). The acrosome is a secretory vesicle in the head of mammalian spermatozoa that is enclosed by a continuous acrosomal membrane. The membrane can be further divided into the inner acrosomal membrane, which is in close proximity to the nuclear membrane, and the outer acrosomal membrane, which is under the plasma membrane that covers the acrosome [65,66] (figure 4). During the AR, the plasma membrane and outer acrosomal membrane fuse, and the acrosomal contents are released. This process uncovers a new set of proteins that will interact with the plasma membrane of the egg during fertilization [66,67] (figure 3). However, once the spermatozoon makes its way through the ZP and reaches the plasma membrane of the egg, the point of contact with the egg is not the tip of the spermatozoon, but rather the equatorial region on either side [8] (figure 4). Thus, it is proteins expressed on these regions, after the acrosome reaction that are probably key to gamete compatibility at this stage of fertilization.

It has historically been thought that the acrosome reaction is triggered when the proteins on the head of the spermatozoa that were exposed during capacitation interact with those on the ZP. Indeed, several studies have shown that the ZP, and ZP3 in particular, have sperm-binding capabilities and can also trigger the AR [68,69]. Additionally, during the AR enzymes are released that can dissolve the ZP, creating a hole through which spermatozoa can pass [70]. However, recent studies have shown that, at least in mice, this is not necessarily the case, and that spermatozoa can undergo the acrosome reaction prior to interaction with the ZP, and even prior to encountering the cumulus cells surrounding the egg [71,72]. Therefore, at this time the trigger(s) for the acrosome reaction and the exact location where it takes place are not known. A role for an interaction with the ZP still seems likely, but what that role is, and how essential it is are now unclear. One possibility is that interaction with the ZP3 may facilitate the completion of the AR, rather than being a key aspect of AR initiation [71].

### 3.2. Summary of gamete interaction stages

In summary, there are two major stages where the proteins of the gametes interact with one another, and thus where the characteristics of these proteins may influence fertilization patterns. First is when the proteins on the head of the spermatozoon interact with those on the egg's zona pellucida. However, as stated above, it was originally thought that it was the proteins exposed during capacitation that interact with the ZP, triggering the acrosome reaction. However, it is now clear that many of the spermatozoa that bind to the ZP have already undergone the AR, and therefore, it is probably proteins exposed on the head post AR that are key to sperm–ZP interactions. Second is when those proteins exposed on the equatorial region of the spermatozoa during the AR interact with those on the egg's plasma membrane (figure 4). By considering which proteins are expressed when, and in what locations, it is possible to identify a suite of potentially interacting candidate genes influencing gametic compatibility.

## 4. Potential gamete compatibility genes

Below is a brief description of the genes that, at the time of this writing, have the most potential for being key players in gamete compatibility. It is divided into those found on each gamete. We reiterate that only one interacting pair is currently known in mammals: Izumo on the sperm, with Juno on the egg. However, the other genes described are known to influence gamete compatibility in some way, even though the details have not yet been worked out. We caution readers that this list represents many of the likely candidates given our current understanding; however, it is not completely exhaustive, and our understanding is still in its infancy. Therefore, some genes that may prove key in the future may not be included here, and some included here may be of limited use.

### 4.1. Sperm

#### 4.1.1. Izumo1

**Figure 5.**
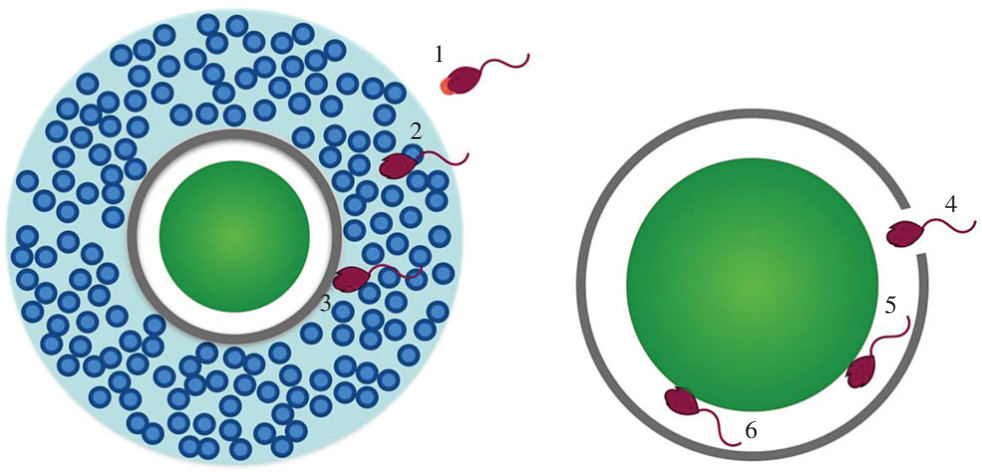
Major steps in fertilization. (1) Spermatozoa undergo the acrosome reaction probably prior to reaching the cumulus mass [71,72]; (2) spermatozoa penetrate the cumulus cells; (3) spermatozoa binds to the zona pellucida; (4) sperm moves through the zona pellucida into the perivitelline space; (5) sperm binds to the egg plasma membrane; (6) sperm fuses with the egg plasma membrane. Note that binding (step 5) and fusion (step 6) are distinct processes, and studies have shown that sperm can bind to the plasma membrane without fusing with it. Figure drawn from [7].

The Izumo1 protein (named after a Japanese marriage shrine) has a large extracellular region, a single transmembrane region and a short cytoplasmic tail [73,74]. During the acrosome reaction, Izumo1 shifts from the anterior head of the sperm to the equatorial segment where fusion takes place [75]. Mice that lack the Izumo1 protein produce normal sperm that are capable of binding to, and penetrating, the zona pellucida, but which are unable to fuse with eggs. The sperm instead built up in the perivitelline space (the space in between the ZP and the plasma membrane of the egg) [76]. An inhibitory antibody bound to this section inhibited sperm–egg fusion but it did not affect sperm motility or egg binding [73]. Binding is a necessary step where the sperm is attached to the egg, before fusion can take place (figure 5) [32]. This suggests the inhibitory effect occurs during the sperm–egg fusion [73]. The putative functional sites where Izumo1 interacts with Juno have been identified, with amino acids 148–163 being particularly important [77,78].

#### 4.1.2. CRISP1

CRISP1, also known as DE (due to showing up on non-denaturing gels as two bands—called proteins D and E [79]) is one member of the cysteine-rich secretory protein (CRISP) family [80]. Members of the CRISP family vary in their biological functions, are found in different mammalian tissues and can even be found in the venom of snakes, lizards and snails [81,82]. The proteins are characterized by 16 conserved cysteine residues, with 10 being clustered in the C-terminal domain [27]. CRISP1 is unique among the candidate genes considered here, in that it appears to be involved in both stages of the sperm–egg interaction. There are two ‘populations’ of CRISP1 expressed on spermatozoa: one loosely bound population that is involved in the initial binding of sperm to the ZP (and which are subsequently released from the sperm during the acrosome reaction); and a second, tightly bound, population that migrates to the equatorial region of the sperm head after the AR and is subsequently involved in egg membrane binding [27]. Mice with a mutated CRISP1 gene were still fertile, but had decreased fusion ability in an environment that promoted sperm competition with healthy sperm [83]. Additionally, the masking of CRISP1 resulted in a significantly lower ability to fertilize eggs that had the cumulus cells and zona pellucida removed. Thus, sperm lacking CRISP1 have a disadvantage in their capacity to both interact with the zona pellucida and fuse with the egg [83]. The egg-binding ability of CRISP1 is located in a specific 12 amino acid sequence known as Signature 2 [84]. However, another member of the CRISP family may compensate in sperm that are lacking CRISP1. CRISP2 may interact with common binding sites on the egg as CRISP1 [85], and CRISP2's Signature 2 region only differs from CRISP1 by two amino acids [83].

#### 4.1.3. CRISP2

CRISP2, also called Tpx-1, is expressed exclusively in male haploid germ cells, and shows high homology (69%) to CRISP1 [85]. Unlike CRISP1, CRISP2 is not involved in ZP binding, and is only associated with binding to the plasma membrane of the egg. CRISP2 is expressed on the equatorial section of the sperm after the acrosome reaction, and experimental studies have found that an inhibitory CRISP2 antibody reduces the percentage of fertilized eggs, with sperm accumulating in the perivitelline space [85]. The antibody had no effect on ZP penetration, sperm motility or the AR [85]. A potential functional site was seen in human males: a polymorphism in exon 9 of CRISP2 resulted in sterility [86].

#### 4.1.4. PKDREJ

PKDREJ is a large, intron-less gene that codes for an approximately 8 kb transcript in humans [87]. Its name is derived from the fact that it has high homology to two different types of genes: the PKD family and the REJ gene. The PKD family of genes code for membrane-bound proteins that form calcium ion channels and are involved in cell–cell and cell–extracellular matrix interactions [88]. A region of PKDREJ is also homologous with the sea urchin REJ gene, which is involved in sperm–egg interaction [89]. The PKDREJ protein is located on the acrosome of the sperm head, suggesting that PKDREJ is involved in ZP binding [90]. Experimental evidence indicates that although PKDREJ is involved in ZP binding, it is not essential [91]. For example, male mice homozygous for a mutated PKDREJ allele could still fertilize eggs, but had lower fertilization success when in a competitive environment with normal sperm. This reduction was due to an increase in the amount of time needed for the acrosome reaction to occur [91]. The likely location of the functionally important segment of the PKDREJ gene is the region homologous to the REJ gene, which corresponds approximately to amino acids 280–800 in humans [25].

#### 4.1.5. PH-20

PH-20 is a plasma membrane protein located on the sperm head as well as on the inner acrosomal membrane, the latter of which appears to be released during the acrosome reaction [92–94]. For many years it was thought that PH-20 is required for sperm binding to the ZP, and this requirement led to investigations of using medicinal blockage of PH-20 as a form of male contraception [92,94]. However, more recent studies suggest that this may not be universal because PH-20-null mice are still fertile [95]. PH-20 appears to have a dual role in fertilization [93,96]. First, PH-20 has enzymatic activity and these proteins covering the head of the sperm are important for penetrating the cumulus layer of cells surrounding the egg. Second, it has a non-enzymatic role in the secondary binding of spermatozoa to the ZP after the acrosome reaction [93,96,97]. The active site of PH-20 required for hyaluronic acid binding, a step in the ability to penetrate the cumulus layer, has been identified at amino acid sites 205–235 [98], but the site required for secondary binding to the ZP has yet to be identified.

#### 4.1.6. Zonadhesin

Zonadhesin is an acrosomal protein that is unique in its ability to bind to the zona pellucida in a species-specific manner [99]. It is localized on the outer acrosomal membrane and exposed during capacitation [100–102]. It differs between species due to rapid evolution and also domain duplication, mRNA splice variation and processing heterogeneity during the functional maturation of the protein [101]. Sperm adhesion to the ZP, or sperm penetration, was decreased when sperm cells were exposed to a zonadhesin antibody [102]. Additionally, mice that lack zonadhesin are fertile, but have lost the species specificity of sperm–ZP fusion. This loss has not been seen with knockout individuals of other sperm proteins [101]. A potential binding region is an exposed fragment of 30 amino acids in MAM (me-prin/A5 antigen/mu receptor tyrosine phosphatase) domain 3 in mice. This section is characterized by a substantially increased rate of positively selected amino acid sites and exhibits high variability in predicted post-translational modifications [103].

### 4.2. Eggs

#### 4.2.1. The zona pellucida

The zona pellucida (ZP) is composed of three different glycoproteins: ZP1, ZP2 and ZP3. In humans and other primates, there is an additional glycoprotein, ZP4 [36]. ZP1 is necessary for forming and maintaining the structural integrity of the zona pellucida [104]. Mice lacking ZP1 still have a zona pellucida, but it is thinner than normal, and has a poorly defined border. This disfiguration can lead to granulosa cells (which make up cumulus cells) accumulating in the perivitelline space, causing functional disorganization within the egg, and resulting in diffusion of the zona matrix. In functional studies of ZP1, ZP1-null mice had reduced fertilization rates (80% of ZP1-null mice were sterile), and those where fertilization was successful had litter sizes that were half those of normal mice [105]. The fact that ZP1-null mice can still be fertile indicates that ZP1 is not essential for proper sperm–egg interaction and fertilization.

In humans, ZP2 is responsible for secondary binding of the acrosome-reacted sperm (following initial binding with ZP3) [106]. ZP2 also provides an effective block to polyspermy. After fertilization, ZP2 is cleaved from the zona pellucida so that additional sperm are unable to bind to the early embryo, ensuring monospermic fertilization [106]. Mice without ZP2 are able to form a thin zona pellucida comprising ZP1 and ZP3. However, the resulting ZP is not sustainable, and the resulting eggs are ZP-free [104]. The absence of the zona pellucida has a negative effect on the development of the egg, resulting in sterility of that female [107].

Although, as stated above, the role of sperm binding with the ZP has been revised with respect to triggering the AR, sperm–ZP binding is still an important step in fertilization, regardless of its role in the AR. For example, mice that lack ZP3 form oocytes without a zona pellucida, which results in sterility [104]. ZP3 is also thought to be responsible for the species-specific binding of sperm to the egg [70]. Although some studies have found indications of which specific regions directly influence sperm binding [108,109], other studies have obtained conflicting results [110,111], and therefore the key regions involved remain unknown. ZP3 polypeptides do not appear to interact with the sperm directly, but rather do so via oligosaccharides that bind to the ZP3 polypeptides [70]. Thus variation within the gene itself, as well as in the associated oligosaccharides, is responsible for the subsequent effects on fertilization. Indeed, previous studies have shown that this gene is under strong selection, causing rapid divergence between species [112].

The role of the human ZP4 is not yet well understood and requires more research. As it is structurally similar to ZP1, it has been assumed that ZP4 also plays a role in maintaining the structural integrity of the human zona pellucida [104].

#### 4.2.2. Juno

Juno is the egg receptor for Izumo1 [29]. Previously called Folr4, this gene was renamed Juno after the Roman goddess of fertility and marriage, once it was recognized as the paired receptor for Izumo. Female mice that lack Juno are completely sterile. Juno is also rapidly shed after fertilization, which could provide an additional block to polyspermy [29]. The shedding of Juno creates a layer of ‘fake’ eggs that could attract and bind acrosome-reacted sperm, preventing them from reaching the already fertilized egg. The interaction of Izumo1 and Juno is a necessary event for adhesion between acrosome-reacted sperm and the egg membrane [29]. Adhesion is the sustained interaction of sperm cells with the egg extracellular matrix that should lead to fertilization with normal sperm [32]. However, these proteins do not facilitate the following step, fusion, which is vital for successful fertilization [29]. Juno has a folate-binding pocket at amino acids 60–175; however, the binding site for Izumo1 has been identified as the surface behind this binding pocket, and specifically within amino acid sites 44–91 and 145–191 [77,78].

#### 4.2.3. CD9

Another putative type of gene for moderating fusion on the egg surface is the tetraspanin family [74]. Tetraspanins are small transmembrane proteins that are thought to affect cell adhesion, motility, proliferation, differentiation and signalling. CD9 is a necessary tetraspanin for gamete fusion [113]. Knockout mice that lack CD9 have severely reduced fertility. The sperm is able to penetrate the ZP and bind to the egg membrane, but the membranes are unable to fuse. The exact role of CD9 in sperm fusion is still unknown. Research suggests that it does not interact directly with a complementary protein on the sperm, but rather it binds with another ‘egg fusion protein’, causing a change in conformation, which then interacts with the sperm [74,113]. The functional sites of CD9 have been identified as part of the large extracellular loop 2, amino acids 173–175. A mutation at these amino acids results in eggs without fusion ability [113].

The genes discussed here primarily code for proteins thought to interact directly with complementary proteins on the other gamete. However, the products of some gamete compatibility genes, such as ZP3, bind to sugar molecules and it is this combined glycoprotein that is involved in gamete interactions [114–116]. This greatly increases the complexity of gamete interactions, with variation in the proteins themselves, the sugar molecules and in the post-translational modification (glycosylation), potentially impacting gamete compatibility. However, we currently know little about the role of glycoproteins in mammalian gamete compatibility, outside of those involved with the ZP, but, given their importance in other taxonomic groups [115], it seems likely that they play an important role, the details of which remain to be discovered.

## 5. Applications

Understanding how characteristics at gamete compatibility genes influence patterns of fertilization has implications for a broad range of fields, ranging from reproductive biology to evolutionary and conservation genetics, to speciation. Here, we will briefly summarize some of these applications, highlighting how they can fill key gaps in our understanding.

In terms of reproductive biology and evolutionary genetics, patterns of non-random fertilization are widespread in nature [117–123]. These are often studied in the context of post-copulatory mate choice, where females are able to ‘choose’ which sperm fertilize their eggs. In species where laboratory experiments are possible, this ‘choice’ appears due to differential fertilization success of different types of sperm relative to the characteristics of each egg [122,123]. Despite the widespread nature of these patterns, however, the mechanisms involved have remained elusive. Thus, identifying the underlying genes and mechanisms has long been regarded as a high priority [118,119,121]. Genes involved in gamete compatibility are clearly the most likely candidate genes influencing these non-random fertilization patterns [14], and their analyses will therefore shed much needed light on the issues of post-copulatory sexual selection, female choice and evolutionary genetics.

Understanding these patterns also has large implications for the fields of conservation biology and conservation genetics. In many of the species where non-random fertilization patterns have been found, fertilizations are biased towards gametes that are genetically dissimilar [117–119,121,123–125]. The result is offspring with higher levels of heterozygosity than expected from a similar-sized random-mating population. In this way, this process can not only slow the decline of heterozygosity expected from genetic drift, but can also maintain heterozygosity at higher levels than expected in small populations. Thus, these biased fertilization patterns can significantly counter the effects of genetic drift, and act to maintain genetic diversity in small populations [126,127]. Moreover, the resulting benefits (primarily offspring with high heterozygosity) have been proposed as one of the main driving forces behind the evolution of polyandry [128–131]. Obtaining a better understanding of how the characteristics of gamete compatibility genes shape fertilization patterns can, therefore, lead to a better understanding of the mechanisms through which patterns of genetic diversity influence reproductive performance and recovery potential in endangered species, and lead to a more thorough understanding of the evolution of different mating systems and strategies.

The process of speciation involves the evolution of reproductive barriers between closely related groups of individuals. Although the development of geographical barriers (resulting in allopatric populations) is often thought to be the trigger for the subsequent development of ‘biological’ barriers, it is the presence of these biological barriers that often underlies where species lines are drawn [132,133]. Gamete compatibility genes are likely candidates for the initial development of reproductive incompatibilities between closely related groups of individuals [17–19,134–136]. Indeed, Gavrilets & Waxman [17] showed that if segregating alleles within a population result in females differing in their compatibility to different males, this will lead to different ‘groups’ of reproductive (compatible) individuals. Over time, this can result in the sympatric evolution of reproductively isolated groups. Thus, gamete compatibility genes are probably a key factor in the evolution of biological reproductive barriers, and provide a clear path through which one species can sympatrically be split into two based solely on different fertilization patterns among existing alleles. A similar process is probably also important in many cases of allopatric speciation with gene flow, where differentiation at gamete compatibility genes underlies the development of reproductive barriers. In this way, the analysis of these genes represents a promising approach for improving our understanding of how genetic characteristics influence the speciation process.

When trying to identify loci influencing specific traits, two approaches are generally used: the candidate gene approach where specific genes or loci are targeted for sequencing and analysis; and genome-wide association studies (GWAS) where tens of thousands of loci (generally single nucleotide polymorphisms, or SNPs) are analysed to screen the genome for regions or loci showing an appropriate signature. The rapid evolution and decreasing cost of methods to characterize and genotype individuals at tens of thousands of SNPs have led to the rapid growth of our understanding of how genotype influences fitness and phenotype based on the GWAS approach [137–139]. However, such studies often involve two stages: the first involving the large-scale genome screening to identify loci with an appropriate signature, and then a second stage of further sequencing and characterization of the area around the SNP originally identified. Therefore, the candidate gene approach may still be a more efficient option in cases where putative candidate genes have been identified [140]. Much research has been conducted on potential gamete compatibility genes, and their likely roles in the fertilization process. Therefore, the goal of this review was to bring this wealth of literature together into one cohesive paper and framework, and to create a list of candidate genes that hold the most potential for success, and therefore serve as a guide for future studies. Moreover, given the broad range, and importance, of processes influenced by gamete compatibility genes, we hope that this paper will serve as motivation for more researchers to pursue this line of inquiry.

## Data accessibility

As a review, this paper did not generate new data of its own. Therefore, the data deposition and accessibility requirements for this journal are not applicable.
